# Comparison of sevoflurane and propofol in combination with remifentanil on the quality of postoperative recovery in patients undergoing laparoscopic bariatric surgery

**DOI:** 10.3389/fmed.2024.1382724

**Published:** 2024-07-24

**Authors:** Zhuolin Shu, Tiancheng Lin, Dingchen Xu, Shuyuan Zheng

**Affiliations:** Department of Anesthesiology, The Sixth People’s Hospital Affiliated to Shanghai Jiao Tong University School of Medicine, Shanghai, China

**Keywords:** sevoflurane, propofol, remifentanil, laparoscopic bariatric surgery, quality of recovery sevoflurane, postoperative recovery quality

## Abstract

**Objective:**

This study aimed to evaluate and compare the effects of sevoflurane + remifentanil (Sev + Rem) and propofol + remifentanil (Pro + Rem) on the postoperative recovery quality of patients undergoing laparoscopic bariatric surgery to determine which anesthesia regimen provides a better overall recovery experience.

**Methods:**

Sixty patients were divided into two groups based on the treatments they underwent: Sev + Rem (*n* = 30) and Pro + Rem (*n* = 30). The Sev + Rem group received sevoflurane inhalation (0.5%, increasing to 0.5–4%) and remifentanil via target-controlled infusion. The Pro + Rem group received propofol [4–8 mg/(kg·h)] and remifentanil via target-controlled infusion. Anesthesia depth was maintained at a bispectral index of 40–60 in both groups. Perioperative data, hemodynamic parameters, and postoperative recovery quality were assessed.

**Results:**

Compared to the Pro + Rem group, the dose of remifentanil in the Sev + Rem group was significantly lower (1693.67 ± 331.75 vs. 2,959 ± 359.77, *p* < 0.001), the proportion of patients used norepinephrine was markedly higher [16 (53.33) vs. 8 (26.67), *p* = 0.035], and the time of extubation was earlier (356.33 ± 63.17 vs. 400.3 ± 50.11, *p* = 0.004). The Hemodynamic results showed the HR in the Sev + Rem group was faster than that in the Pro + Rem group at the beginning of surgery and 1 h post-surgery (67.37 ± 4.40 vs. 64.33 ± 4.44, *p* = 0.010, 69.07 ± 4.23 vs. 66.40 ± 5.03, *p* = 0.030). In regard to the assessment of postoperative recovery quality, the emotional state scores in the Sev + Rem group were significantly lower than the Pro + Rem group (36.83 ± 2.79 vs. 39.50 ± 4.64, *p* = 0.009).

**Conclusion:**

The two anesthesia modalities (Sev + Rem and Pro + Rem) have their advantages and disadvantages for patients undergoing laparoscopic bariatric surgery and have comparable effects on postoperative recovery quality.

## Introduction

In recent years, the proportion of obese patients has increased due to unbalanced diets despite improved living standards. Over 1 million people in China die annually from obesity-related complications, including diabetes, cardiovascular disease, and cerebrovascular disease (1, 2), and obesity prevalence is projected to reach 51% by 2030 (3). Obesity can be classified by BMI based on the following: 25–29.9 kg/m^2^ for pre-obesity (overweight), 30.0–34.9 kg/m^2^ for obesity class I, 35.0–39.9 kg/m^2^ for obesity class II, and ≥ 40 for extreme obesity (4). Patients with pre-obesity and obesity class I can be treated with diet control, exercise, and medication (5), and bariatric surgery is recommended for severe obesity and its complications. However, surgery is not advised for those with unrealistic expectations, unwilling to accept surgical risks, or unable to follow postoperative dietary and lifestyle changes (5, 6).

Patients with severe obesity tend to have decreased lung compliance and hypoventilation syndrome, as well as being prone to the accumulation of fat-soluble anesthetics due to excessive fat content (7). Therefore, the incidence of postoperative anesthesia adverse events and complications is significantly increased in patients with severe obesity compared with other patients (8, 9). Besides, the selection of appropriate anesthetic drugs and modalities is particularly important for patients undergoing bariatric surgery. Thiopental sodium or propofol has been reported to be commonly used for anesthesia induction and remifentanil for anesthesia maintenance before bariatric surgery (10). Remifentanil, a μ-type receptor agonist, can reach blood–brain equilibrium within 1 min after injection, with rapid onset and short maintenance time. In addition, the analgesic effect of remifentanil depends on its dosage and it does not accumulate in the body even after prolonged or repeated injections (11). Sevoflurane is a halogenated inhalational anesthetic. In addition to the stable response of the patient to the drug during the induction period, the effect on intraoperative hemodynamics is little, and the postoperative recovery is rapid and thorough (12). Propofol has a rapid onset and a short induction time. However, the effect of propofol is more significant on the hemodynamic indexes and the stress response is increased. Such a drug adversely affects the quality of postoperative recovery (13).

At present, studies on the prognosis of patients with two different anesthetic modalities [sevoflurane-remifentanil (Sev + Rem) and propofol-remifentanil (Pro + Rem)] in other surgeries have been reported, such as Pro + Rem plays the protective effect on endothelial glycocalyx in the deep inferior epigastric artery perforator flap breast reconstruction; the patients in the Pro + Rem group recover quickly after electroconvulsive therapy; the hemodynamics are stable; and there is no difference between Pro + Rem and Sev + Rem in postoperative analgesia after shoulder arthroscopy (14–16). Nevertheless, it is unknown whether Pro + Rem and Sev + Rem have different effects on the quality of postoperative recovery in laparoscopic metabolic and bariatric surgery patients. Therefore, this study intends to compare the effects of the above two anesthetic modalities on the quality of postoperative recovery in patients undergoing laparoscopic bariatric surgery, thereby providing a reference for the anesthesia strategy for such patients.

## Materials and methods

### Study design and sample size calculation

This study was designed to evaluate the effects of two anesthesia regimens on postoperative recovery in patients undergoing laparoscopic bariatric surgery. A sample size of 60 patients was chosen based on preliminary power analysis, which indicated that this number would provide sufficient power (80%) to detect a clinically significant difference in recovery quality between the two groups, assuming a medium effect size and a significance level (alpha) of 0.05. This sample size was selected to ensure the study could differentiate between the effects of the two anesthesia regimens and to minimize the risk of the study being underpowered.

### Inclusion criteria

Patients diagnosed with obesity undergoing bariatric surgery in our hospital were selected based on the following inclusion criteria: (1) the patients were aged 18–65 years; (2) the patients with a BMI ≥35 kg/m^2^ met the indications for bariatric surgery; (3) the patients of the American Society of Anesthesiologist (ASA) physical status were II–III (17); and (4) the patient participated in the trial voluntarily and signed the informed consent form. The exclusion criteria included: (1) preoperative liver and renal dysfunction; (2) preoperative presence of chronic pain and long-term use of opioid painkillers; (3) congenital heart disease, arrhythmia, or hypertension; (4) allergy to the drugs used in the trial; and (5) presence of contraindications to laparoscopic surgery. The study complied with the Declaration of Helsinki and was approved by the Ethics Committee of The Sixth People’s Hospital Affiliated to Shanghai Jiao Tong University School of Medicine.

### Surgery and anesthesia modalities

In this study, the patients were divided into the Sev + Rem group and the Pro + Rem group based on the treatments they underwent. Patients in both groups underwent laparoscopic bariatric surgery, including gastric sleeve and Roux-en-y gastric bypass. All patients were informed to fast for 8 h and deprived of water for 4 h prior to surgery. Before anesthesia, venous access to the upper extremities was obtained, electrocardiogram monitoring and depth of anesthesia monitoring were connected, and oxygen masks were provided. The patients in the Sev + Rem group were induced with sevoflurane, starting at a concentration of 0.5% and gradually increasing by 0.5–4% with each breath. With the increase in the number of breaths, the concentration gradually increased by 0.5–4% (18). The concentration was elevated every 2–3 breaths. Besides, the oxygen flow was set to 4 L/min. Rocuronium bromide (0.8 mg/kg) was administered intravenously after loss of consciousness, and the patients were mechanically ventilated by tracheal intubation with a tidal volume of 10 mL/kg. Remifentanil (4.0 ng/mL at the site of action) was injected continuously, and sevoflurane inhalation was maintained. The patients in the Pro + Rem group were given propofol at 4–8 mg/(kg·h) and a target-controlled infusion of remifentanil was administered, with the blood drug concentration at 3 ng/mL (18, 19). Anesthesia was stopped after surgery. During surgery, norepinephrine was administered to manage hypotension. It was diluted in a standard solution of 0.1 mg/mL and titrated to maintain mean arterial pressure (MAP) within 65–75 mmHg. The dosing was adjusted based on the hemodynamic response of each patient, with continuous monitoring to ensure safety and efficacy. The depth of anesthesia in all patients was determined using the bispectral index (BIS), of which the BIS was maintained at 40–60.

### Perioperative data

Perioperative data of patients were collected, including baseline data such as age, sex, weight, height, BMI, ASA class (II/III), smoking history (yes/no), alcohol abuse history (yes/no); and intraoperative and postoperative data such as surgical method, remifentanil dose, vasoactive drugs use, blood loss, duration of anesthesia, operation time, extubation time, post-anesthesia care unit (PACU) length of stay, onset time of postoperative off-bed activity, time of first postoperative defecation, and hospitalization time.

### Hemodynamic testing

Mean arterial pressure (MAP) and heart rate (HR) were measured before the induction of anesthesia (T1), after the induction of anesthesia (T2), at the beginning of surgery (T3), 1 h after surgery (T4), and at the end of surgery (T5).

### Assessment of quality of recovery

In this study, the quality of recovery-40 (QoR-40) questionnaire was used to assess the quality of recovery of patients in both groups at 24 h after surgery. The QoR-40 questionnaire measures 40 items in five dimensions: physical comfort, physical independence, emotional state, psychological support, and pain. Each item is scored from 1 to 5, with a total score of 200 (optimal quality) (20).

### Anesthesia-related adverse reactions

The adverse reactions such as nausea, vomiting, post-extubation dyspnea, fever and chills, hypoxemia, and bradycardia were recorded from the end of anesthesia to the end of awakening.

### Study endpoints

The study’s primary endpoint was the quality of recovery, assessed using the QoR-40 questionnaire at 24 h post-surgery, focusing on physical comfort, physical independence, emotional state, psychological support, and pain. Secondary endpoints included intraoperative and postoperative metrics (surgical method, remifentanil dose, vasoactive drug use, blood loss, duration of anesthesia, operation time, extubation time, PACU length of stay, onset time of postoperative off-bed activity, time of first postoperative defecation, and hospitalization time). Additionally, hemodynamic measurements, including heart rate and mean arterial pressure, were recorded at five time points (before induction of anesthesia, after induction, at the beginning of surgery, 1 h after surgery, and at the end of surgery). The incidence of anesthesia-related adverse reactions, such as nausea, vomiting, post-extubation dyspnea, fever and chills, hypoxemia, and bradycardia, were also monitored from the end of anesthesia to the end of awakening. These endpoints aimed to compare the effects of sevoflurane vs. propofol, both combined with remifentanil, on postoperative recovery quality and various perioperative parameters in patients undergoing laparoscopic bariatric surgery.

### Statistical analysis

Measurements data of normal distribution were expressed as mean ± standard deviation. Independent samples *t*-test was used to compare the means between the two groups. Enumeration data were represented as percentages and rates, and the chi-square was used to compare the groups. For all statistical comparisons, *p* < 0.05 was considered statistical significance. Statistical analysis and plotting were performed using GraphPad Prism 8.0.

## Results

### Comparison of baseline data of patients

A total of 60 patients were included in this study, including 33 males and 27 females. The mean age of patients in the Sev + Rem group was 48.53 ± 8.03 years old, with 18 males and 12 females; the mean age of patients in the Pro + Rem group was 49.57 ± 6.31 years old, with 15 males and 15 females. Notably, there was no statistical difference in age and sex ratio between the two groups and also no statistically significant difference in height (1.71 ± 0.08 vs. 1.71 ± 0.09 m), weight (108.3 ± 12.18 vs. 108.73 ± 12.19 kg), BMI (37.09 ± 1.64 vs. 36.88 ± 1.18 kg/m^2^), ASA class, and previous history of smoking and alcohol in the below groups (Table 1).

**Table 1:** Comparison of baseline data of patients in Sev + Rem and Pro + Rem groups.

Characteristics	Sev + Rem group (*n* = 30)	Pro + Rem group (*n* = 30)	*X*^2^/*t*	*p*
Mean age	48.53 ± 8.03	49.57 ± 6.31	−0.554	0.582
Sex (%)			0.606	0.436
Male	18 (60.00)	15 (50.00)		
Female	12 (40.00)	15 (50.00)		
Height (m)	1.71 ± 0.08	1.71 ± 0.09	−0.366	0.716
Weight (kg)	108.3 ± 12.18	108.73 ± 12.19	−0.138	0.891
BMI (kg/m^2^)	37.09 ± 1.64	36.88 ± 1.18	0.562	0.576
ASA class (%)			0.317	0.573
II	8 (26.67)	10 (33.33)		
III	22 (73.33)	20 (66.67)		
Smoking history (%)			0.635	0.426
No	20 (66.67)	17 (56.67)		
Yes	10 (33.33)	13 (43.33)		
Alcohol abuse history (%)			0.268	0.605
No	17 (56.67)	15 (50.00)		
Yes	13 (43.33)	15 (50.00)		

BMI, Body mass index; ASA, American Society of Anesthesiologist.

### Comparison of perioperative data

There was no statistical difference in the choice of surgical methods between the two groups. In terms of anesthetic drug use, the dose of remifentanil in the Sev + Rem group (1693.67 ± 331.75 μg) was significantly lower than that in the Pro + Rem group (2,959 ± 359.77 μg) (*p* < 0.001). No significant difference in the use of remaining vasoactive drugs was seen between the two groups, except for the higher use of norepinephrine in the Sev + Rem group [16 (53.33) vs. 8 (26.67), *p* = 0.035]. In terms of postoperative monitoring, the time of tracheal extubation was earlier in the Sev + Rem group compared to the Pro + Rem group (356.33 ± 63.17 vs. 400.3 ± 50.11, *p* = 0.004). There was no significant difference between the two groups in intraoperative blood loss, duration of anesthesia, operative time, PACU length of stay, onset time of postoperative off-bed activity, time of first postoperative exhaust and defecation, and hospitalization time (Table 2).

**Table 2:** Comparison of perioperative data in Sev + Rem and Pro + Rem groups.

Factors	Sev + Rem group (*n* = 30)	Pro + Rem group (*n* = 30)	*X*^2^/*t*	*p*
Surgical procedures (%)			0.606	0.436
Gastric sleeve	18 (60.00)	15 (50.00)		
Roux-en-y gastric bypass	12 (40.00)	15 (50.00)		
The dose of remifentanil (μg)	1693.67 ± 331.75	2,959 ± 359.77	−14.162	0.000
The use of vasoactive drugs (%)				
Ephedrine (%)	11 (36.67)	13 (43.33)	0.278	0.598
Norepinephrine (%)	16 (53.33)	8 (26.67)	4.444	0.035
Urapidil (%)	19 (63.33)	16 (53.33)	0.617	0.432
Atropine (%)	24 (80.00)	22 (73.33)	0.373	0.542
Blood loss (mL)	163.33 ± 50.54	149.00 ± 46.86	1.139	0.259
Duration of anesthesia (min)	565.87 ± 73.18	581.47 ± 67.00	−0.861	0.393
operative time (min)	518.70 ± 72.81	526.43 ± 58.12	−0.455	0.651
The time of tracheal extubation (min)	356.33 ± 63.17	400.3 ± 50.11	−2.987	0.004
PACU length of stay (min)	5.93 ± 1.55	5.67 ± 2.12	0.555	0.581
Onset time of postoperative off-bed activity (h)	13.23 ± 1.70	13.97 ± 2.11	−1.484	0.143
Time of first postoperative exhaust (h)	27.60 ± 2.08	27.90 ± 2.62	−0.492	0.625
Time of first postoperative defecation (h)	33.17 ± 3.03	32.00 ± 2.79	1.551	0.126
Hospitalization time (d)	7.00 ± 0.69	7.03 ± 0.67	−0.189	0.850

### Hemodynamic monitoring

The hemodynamics of the patients were monitored before T1, T2, T3, T4, and T5. This study revealed that the HR of the two groups showed a downward trend after T1; the HR was markedly lower in the Pro + Rem group in comparison with the Sev + Rem group at T3 and T4 (67.37 ± 4.40 vs. 64.33 ± 4.44, *p* = 0.010); and the HR was almost the same in both groups at T5. The MAP in the Pro + Rem group dropped slightly as opposed to the Sev + Rem group after T2. Notably, no statistically significant difference was observed in MAP between the two groups at each time point (Table 3).

**Table 3:** Comparison of hemodynamics between the Sev + Rem group and the Pro + Rem group.

Variables	Grouping	T1	T2	T3	T4	T5
HR	Sev + Rem group	75.27 ± 4.93	70.17 ± 5.66	67.37 ± 4.40	69.07 ± 4.23	70.43 ± 5.87
	Pro + Rem group	75.07 ± 4.57	68.27 ± 5.36	64.33 ± 4.44	66.40 ± 5 0.03	69.53 ± 5.35
	*t*	0.163	1.334	2.657	2.222	0.620
	*p*	0.871	0.187	0.010	0.030	0.537
MAP	Sev + Rem group	90.40 ± 7.16	84.17 ± 5.95	81.30 ± 5.47	81.67 ± 5.92	83.23 ± 6.58
	Pro + Rem group	90.50 ± 5.12	83.27 ± 7.07	79.57 ± 6.00	80.37 ± 6.86	82.70 ± 6.31
	*t*	−0.062	0.533	1.170	0.786	0.320
	*p*	0.951	0.596	0.247	0.435	0.750

T1, Before the induction of anesthesia; T2, After the induction of anesthesia; T3, At the beginning of surgery; T4, 1 h after surgery; T5, At the end of surgery; HR, Heart rate; MAP, Mean arterial pressure.

### Assessment of quality of recovery

After evaluating the QoR-40 questionnaire in the two groups at 24 h after surgery, the study suggested that the score of postoperative emotional state in the Pro + Rem group was higher than that in the Sev + Rem group (39.50 ± 4.64 vs. 36.83 ± 2.79, *p* = 0.009). Besides, the difference was not statistically significant between the two groups in the remaining aspects of the assessment. The total scores of QoR-40 questionnaires in the Sev + Rem group and the Pro + Rem group were almost the same, indicating that there was no significant difference in the postoperative quality of recovery between the two groups (Table 4).

**Table 4:** Comparison of the postoperative quality of recovery between the Sev + Rem group and the Pro + Rem group.

Variables	Sev + Rem group (*n* = 30)	Pro + Rem group (*n* = 30)	*t*	*p*
Physical comfort	48.63 ± 4.89	47.37 ± 5.60	0.933	0.354
Physical independence	12.83 ± 2.31	12.13 ± 2.00	1.257	0.214
Emotional state	36.83 ± 2.79	39.50 ± 4.64	−2.698	0.009
Psychological support	30.30 ± 4.40	30.07 ± 4.14	0.211	0.833
Pain	29.37 ± 3.35	28.33 ± 3.92	1.099	0.276
Total scores	157.97 ± 8.07	157.40 ± 8.63	0.263	0.794

### Anesthesia-related adverse reactions

The common adverse reactions of patients after anesthesia were recorded to investigate the effects of different anesthesia modalities on the postoperative anesthesia-related adverse reactions of patients. As shown in Table 5, nausea was the most common adverse reaction after anesthesia in this study, with a total incidence of 63.33%, while the incidence of fever and chills was the lowest, both 5%. However, different anesthesia modalities did not affect the incidence of postoperative anesthesia-related adverse reactions in patients, and no statistically significant difference was found in adverse reactions between the two groups.

**Table 5:** Comparison of postoperative anesthesia-related adverse reactions between the Sev + Rem group and Pro + Rem group.

Variables	Sev + Rem group (*n* = 30)	Pro + Rem group (*n* = 30)	*X* ^2^	*p*
Nausea (%)	18 (60.00)	20 (66.67)	0.287	0.592
Vomiting (%)	5 (16.67)	3 (10.00)	0.144	0.704
Post-extubation Dyspnea (%)	2 (6.67)	4 (13.33)	0.185	0.667
Fever (%)	2 (6.67)	1 (3.33)	0.000	1.000
Chills (%)	1 (3.33)	2 (6.67)	0.000	1.000
Hypoxemia (%)	3 (10.00)	4 (13.33)	0.000	1.000
Bradycardia (%)	3 (10.00)	6 (20.00)	0.523	0.470

## Discussion

In this study, the two anesthesia modalities (Sev + Rem and Pro + Rem) have their respective advantages and disadvantages for patients undergoing laparoscopic bariatric surgery with comparable effects on the quality of postoperative recovery. Sev + Rem was associated with comparatively more stable intraoperative heart rates, shorter extubation times, and reduced doses of remifentanil but had higher proportion of patients used norepinephrine, which resulted in lower postoperative emotional state scores. Pro + Rem improved the postoperative emotional state and reduced remifentanil doses but led to longer extubation times and greater fluctuations in heart rate. Further studies with larger sample sizes and extended follow-up periods are needed to draw more definitive conclusions about the optimal anesthesia regimen for this patient population.

Patients with severe obesity have a difficult airway, decreased lung compliance and reduced lung function before surgery. High airway pressure mechanical ventilation during anesthesia can worsen lung function, posing perioperative risks (20). In this demographic, sevoflurane improves pulmonary ventilation, protects lung function, and allows rapid recovery of consciousness with minimal secondary distribution (21, 22), thereby aiding in lung function recovery (23). In our present study, the advantages of sevoflurane for respiratory function recovery were demonstrated, suggesting that Sev + Rem is more conducive to early tracheal extubation than Pro + Rem. Sevoflurane can also protect cardiomyocytes under stress by reducing oxygen-free radical generation during hypoxia (24). Additionally, Sev + Rem can inhibit catecholamine production and effectively relax the vascular endothelium, protecting cardiomyocytes by producing prostaglandins and nitric oxide (21). The higher proportion of patients used norepinephrine in the Sev + Rem group may be related to the vasodilating effect of sevoflurane, but it did not affect the quality of postoperative recovery. Propofol provides fast, smooth anesthesia, but its limited analgesic effect can impact the circulatory system, affecting HR (22). Herein, we found that HR fluctuations were more pronounced in the Pro + Rem group than in the Sev + Rem group. Remifentanil, a μ-opioid receptor agonist, is an effective analgesic that can reduce intraoperative stress responses and correct hemodynamic abnormalities caused by surgery (23). In this study, we found that the combination of Sev + Rem provides synergistic effects (24). Therefore, Sev + Rem is effective in maintaining intraoperative circulatory stability in patients undergoing laparoscopic weight loss surgery.

A better postoperative emotional state was observed in the Pro + Rem group. Propofol has been shown to elevate extracellular dopamine levels, inhibit dopamine reuptake, and increase glutamate levels (an excitatory neurotransmitter), thereby reducing negative emotions and enhancing positive emotions such as positivity and enthusiasm (25). Conversely, sevoflurane is associated with postoperative anxiety, depression, and cognitive dysfunction (26, 27). Although emotional recovery differed between the groups, their total scores for overall recovery were similar. Thus, the overall advantage was not evident in the Pro + Rem group. However, it should be noted that improvements in postoperative emotional state observed in the Pro + Rem group could be linked to propofol’s known effects on dopamine and glutamate, though this interpretation is tentative given the study’s limited sample size of 60 patients. Additionally, sevoflurane has been found to have a higher incidence of postoperative nausea and vomiting, immunosuppression, respiratory-related adverse effects, and cognitive deficits than those of propofol (28, 29). However, our study did not find statistically significant differences in postoperative anesthesia-related adverse effects between the two groups, which contradicts previous findings. This discrepancy might be due to the small sample size and the selection of middle-aged patients around 48 years old, who may have better tolerance for anesthetic adverse reactions.

There were some limitations in this study. First, the relatively small sample size and single-center design limit the generalizability of the findings. Future studies should involve larger cohorts and multiple centers to enhance the robustness and applicability of the results. Second, the short-term follow-up of 24 h postoperatively does not capture mid-and long-term outcomes. Extended follow-up periods are needed to assess delayed adverse effects and longer-term recovery trajectories. Additionally, the study population consisted primarily of middle-aged patients with relatively good health, aside from obesity, which limits applicability to older adults or those with multiple comorbidities. Future studies should include more diverse populations.

In conclusion, this study showed that the two anesthesia modalities, Sev + Rem and Pro + Rem, have their respective advantages and disadvantages for patients undergoing laparoscopic bariatric surgery, with comparable effects on the quality of postoperative recovery. Sev + Rem can reduce the dose of remifentanil, shorten tracheal extubation time, and maintain intraoperative heart rate more effectively. However, it was also associated with higher proportion of patients used norepinephrine and lowered the postoperative emotional state. Pro + Rem improves the postoperative emotional state and is associated with reduced dosing of remifentanil, with shorter time to extubation, and with reduced variability in intraoperative heart rate.

## Data availability statement

The raw data supporting the conclusions of this article will be made available by the authors, without undue reservation.

## Ethics statement

The studies involving humans were approved by Ethics Committee of the Sixth People’s Hospital Affiliated to Shanghai Jiao Tong University School of Medicine. The studies were conducted in accordance with the local legislation and institutional requirements. The participants provided their written informed consent to participate in this study.

## Author contributions

ZS: Conceptualization, Writing – original draft. TL: Formal Analysis, Writing – review & editing. DX: Formal Analysis, Writing – review & editing. SZ: Conceptualization, Writing – original draft.
